# Adipokines as potential biomarkers for type 2 diabetes mellitus in cats

**DOI:** 10.3389/fimmu.2022.950049

**Published:** 2022-09-30

**Authors:** Olga Sierawska, Paulina Niedźwiedzka-Rystwej

**Affiliations:** ^1^ Doctoral School, University of Szczecin, Szczecin, Poland; ^2^ Institute of Biology, University of Szczecin, Szczecin, Poland

**Keywords:** feline, diabetes mellitus, adipokines, leptin, adiponektin

## Abstract

Type 2 diabetes mellitus (T2DM) is no longer only a disease of humans, but also of domestic animals, and it particularly affects cats. It is increasingly thought that because of its unique characteristics, T2DM may belong not only to the group of metabolic diseases but also to the group of autoimmune diseases. This is due to the involvement of the immune system in the inflammation that occurs with T2DM. Various pro- and anti-inflammatory substances are secreted, especially cytokines in patients with T2DM. Cytokines secreted by adipose tissue are called adipokines, and leptin, adiponectin, resistin, omentin, TNF-α, and IL-6 have been implicated in T2DM. In cats, approximately 90% of diabetic cases are T2DM. Risk factors include older age, male sex, Burmese breed, presence of obesity, and insulin resistance. Diagnosis of a cat requires repeated testing and is complicated compared to human diagnosis. Based on similarities in the pathogenesis of T2DM between humans and cats, adipokines previously proposed as biomarkers for human T2DM may also serve in the diagnosis of this disease in cats.

## 1 Introduction

Chronic low-grade inflammation is common in pathologies such as obesity, cancer, type 2 diabetes (T2DM), and cardiovascular diseases (1). The immune system is usually described in terms of its protective function against infections and its role in the development of autoimmunity. In contrast, interactions between the immune system and metabolic processes have been reported for more than a decade, resulting in a field of research termed immunometabolism. So far, it has been shown (2) that the development of metabolic diseases can be linked to inflammation, which in some cases reflects the activation of immune responses. There are many evolutionarily conservative interactions between the immune response and metabolism. Proper maintenance of this delicate balance is critical for health and has important implications for many pathological conditions such as obesity, diabetes mellitus, and other chronic non-communicable diseases (3). Metabolic dysregulation results in immune cell involvement. If the organism is obese, pro-inflammatory M1 macrophages make up the majority of the macrophage population in adipose tissue, with more anti-inflammatory M2 in lean individuals. They secrete pro-inflammatory cytokines (TNF-α, IL-6, IL-1β, IL-12, and MCP-1), which not only have a role in promoting insulin resistance but also recruit other immune cells to the site of inflammation (4). Among the cells recruited are neutrophils, lymphocytes, and innate lymphoid cells (ILC), including natural killer (NK) cells, mast cells, and eosinophils (4, 5). Elevated levels of neutrophil, have been observed in both obese humans and mouse models fed a high-fat diet (HFD), and are therefore thought to be involved in modulating adipose tissue inflammation in the early stages of obesity (6). Lymphocytes are recruited to the site of inflammation by specific cytokines. In obesity, reduced levels of CD4 + CD25 + Foxp3+ lymphocytes are observed, indicating their depletion, which contributes to the development of insulin resistance. In contrast, CD8 T-lymphocyte levels increase in adipose tissue as early as 2 weeks of HFD feeding, and levels are high in obesity. The important role of CD8 T lymphocytes in the development of inflammation lies in their ability to induce the differentiation of macrophages from monocytes. B lymphocytes influence adipose tissue inflammation by modulating T lymphocyte activity (7). Mast cells have also been shown to be involved in the regulation of adipose tissue in obesity. Eosinophils, on the other hand, induce a faster rate of macrophage differentiation into the M2 phenotype by secreting the cytokines IL-4 and IL-13, which have an anti-inflammatory effect on adipose tissue inflammation (5).

The site of many inflammatory processes is in adipose tissue. Adipose tissue is classified as connective tissue, as a specialized tissue composed mainly of adipocytes (fat cells). Adipocytes are composed of a single, large lipid droplet with a thin envelope of cytoplasm between it and the plasma membrane (3). Currently, it is widely agreed that adipose tissue is a strongly active and complex hormonal and metabolic gland consisting of adipocytes, connective tissue matrix, neural tissue, stromal cells, and immune cells (8). The link between adipose tissue metabolism and the immune system is indicated by the fact that immune responses often lead to loss of body weight, and in obese individuals, the immune system malfunctions, causing them to suffer an increased incidence and severity of infectious diseases (9, 10). In obese individuals, only half the number of the monocytes mature into macrophages, compared to patients without obesity (10). In addition, macrophages contribute to the adipose organ in the generation of inflammation (11). Animal studies also yield similar data. Decreased immunocompetence, decreased activity of cellular immune components, decreased resistance to infection, and decreased macrophage killing capacity are observed in obese individuals (10).

Studies suggest (2) that T2DM, like type 1 diabetes (T1DM), may belong to the autoimmune diseases, and the two types of diabetes may have more in common than previously thought (3). In T2DM with obesity, adipocyte size is increased. This alters the levels of circulating pro-inflammatory cytokines contributing to the recruitment of immune cells to the site of inflammation, which is adipose tissue (12). Additionally, the insulin resistance in T2DM may be the result of attacks by immune system cells that are designed to produce antibodies that fight pathogens on body tissues (3). Although T2DM has been classified as a metabolic disease for more than 30 years, autoimmunity in T2DM is a growing topic (13). Chronic inflammation induced by obesity, a major causative factor in T2DM, affects the activation of innate and adaptive immunity, including the secretion of pro-inflammatory cytokines that activate T lymphocytes, B lymphocytes and macrophages. As a result of the strong impact of inflammatory and autoimmune reactions, β-cells undergo apoptosis, and impaired insulin secretion in β-cells affects the development of hyperglycemia (13, 14). Another important feature of autoimmunity in T2DM is impaired B lymphocyte function. People with T2DM do not secrete the anti-inflammatory cytokine, interleukin-10, and they secrete a much higher amount of pro-inflammatory cytokines (13, 14). Patients with T2DM have reduced resistance to infection, which may be due to the reduced ability of B lymphocytes to produce de novo antibodies (14). Additionally, T lymphocytes are an important aspect in autoimmunity in T2DM. Self-reactive T lymphocytes are in decline in patients with T2DM (13). T-cell receptors (TCRs), which are designed to recognize antigen and are differentially expressed in healthy individuals, are limited in T2DM patients (15). T-lymphocytes, together with macrophages, by releasing pro-inflammatory cytokines, contribute to increased pancreatic islet inflammation (13). A role for dysbiosis of the gut microbiota in toll-like receptor (TLR) activation in adipocytes and production of pro-inflammatory adipokines is also indicated (15). Current immunomodulatory therapies in T2DM target IL-1β, block the NF-kB pathway and increase circulating adiponectin with positive results (13).

Because of differences in the structure of adipose tissue in rodents, the most popular model organism for humans, research on it is not fully developed. Murine adipocytes differ in structure from those of humans: mouse adipocytes contain many fat droplets, while human adipocytes contain one (3). There are differences in adipogenesis too. Additionally, molecular differences adversely affect the use of mouse models: in mice, TNF-α is a major factor in haptoglobin expression, and in humans, it induces downregulation of haptoglobin mRNA (16). Cats, unlike rodents, develop all the symptoms of T2DM: insulin resistance, impaired β-cell function and reduced numbers of them, and pancreatic amyloid deposition (17).

## 2 T2DM in cats

In cats, T2DM is the most common among the various types of diabetes, with about 90% of diabetes cases being T2DM (18). By comparison, in dogs, most cases of diabetes are type 1 diabetes (19). T2DM is characterized by insulin resistance and decreased insulin secretion due to pancreatic β-cell failure (18, 20). Insulin resistance is differentiated by impaired tissue response to insulin and can lead to hyperglycemia and excessive pancreatic insulin secretion leading to balance (20). Although T2DM in cats is the most common type of DM and is very similar to human T2DM, unlike in humans, treatment may include insulin therapy (21). This is because DM in cats has been proposed to be divisible into insulin-dependent DM (IDDM) and non-insulin-dependent diabetes mellitus (NIDDM), and T2DM in cats qualifies as IDDM (22). In this paper, however, we will use the term T2DM and note that it is a type of IDDM in cats.

### 2.1 Risk factors

Risk factors for developing T2DM in cats include both genetic and environmental conditions, like advancing age, male sex (including being neutered), genetic aspects (breed), diet-dependent obesity and physical inactivity (18, 21, 23, 24).

#### 2.1.1 Age

The probability of cats developing DM has been shown to increase beyond the age of 6 years, and it is highest at 11-13 years (25–28). This may be because insulin sensitivity and β-cell functionality decrease with age, and amyloid deposition in the pancreas may be high (26).

#### 2.1.2 Sex

An increased risk of developing T2DM has been observed in male cats. The more their body weight increased, the higher the risk was. Interestingly, this pattern did not occur in female cats — despite increasing body weight, there was no significant increase in the risk of developing T2DM (25). This may be because male cats may be naturally more insulin resistant than female cats. Additionally, the mere fact of being a female purebred cat rather than a mixed-breed cat was protective against developing T2DM (25). Males, regardless of breed, were at the same risk of developing the disease (25). In males, neutering also appears to have an impact on the development of T2DM disease, as they are 2-9 time more likely to develop the disease then uncastrated males (21, 29). After neutering there are changes in hormone levels: an increase in prolactin, leptin, and insulin-like growth factor 1 (IGF-1), and a decrease in testosterone and estrogen. Among estrogens, estradiol has been shown to regulate normal food intake. In neutered males, estradiol levels are significantly reduced, by up to half in plasma (30). Therefore, especially in males, ad libitum access to food is a large risk factor for the development of obesity and resulting T2DM. The exceptions are Burmese cats, in which the gender factor does not affect the chances of developing T2DM — both male and female cats are equally susceptible to the disease (29).

#### 2.1.3 Breed and other genetic factors

Remarkably, there is one breed that is particularly prone to developing diabetes: the Burmese cat (22, 23, 31). Type 2 diabetes is up to 4 times more common in Australian-bred Burmese cats (32), and the New Zealand population is also at increased risk (33). This is probably due to the presence of an inborn metabolic deficiency in certain of these cats. This results in postprandial hypertriglyceridemia after high-fat meals. In Burmese cats with postprandial hypertriglyceridemia, lipoprotein lipase (LPL) deficiency and elevated very-low-density lipoprotein (VLDL) concentrations have been observed (31). This may be because the breed was developed from a small number of individuals and the propensity for the disease is passed down generationally. A genetic factor is also indicated by the fact that USA-bred Burmese cats, a population quite isolated from others because Burmese breeders in Australia, New Zealand, and the United Kingdom have not approved Burmese cats from the USA for more than 20 years, do not suffer from an increased risk of developing T2DM (34). With further genetic studies of Burmese cats, it would be possible to locate the alleles responsible for the increased risk of developing type 2 diabetes in cats.

In addition to the Burmese breed, high incidence rates of DM risk were also found in the Russian blue, Norwegian forest cat, European shorthair, and Abyssinian breeds (27).

Additionally, in cats mutations in the sequence of the gene encoding the melanocortin 4 receptor (MC4-R), decreased expression levels of the insulin receptor substrate 1 (IRS-1), insulin receptor substrate 2 (IRS-2) and phosphoinositide 3-kinase (PI3-K) genes, or decreased expression levels of the gene encoding the glucose transporter type 4 (GLUT4) in muscle and adipose tissues have also been observed (21). As in cats, in humans genetic factors are also important in the pathogenesis of T2DM (33). A mutation in the gene encoding the melanocortin 4 receptor (MC4-R) causing it to be defective in humans is one of the most common causes of obesity, glucose intolerance and T2DM (35, 36). MC4-R inhibition leads to increased fat cell accumulation (37). Polymorphisms in the IRS-1 gene in humans are associated with an increased risk of developing gestational diabetes, and the gene itself is involved in the development of insulin resistance in T2DM (38). Notable among the polymorphisms in IRS-1 affecting the development of T2DM and insulin resistance are IRS-1 substitutions by Gly to Arg 972 (39). IRS-2 also plays an important role in the initiation and progression of T2DM. It has been shown that increasing its expression in patients with insulin resistance can positively delay the development of diabetes (40), and its reduced expression can induce β-cell apoptosis (41). PI3-K is involved in the IRS substrate pathway, which is impaired by insulin inefficiency and hyperglycemia (42). GLUT4 is involved in glucose uptake in muscle and adipose tissues, and changes in its expression affect glycemic control in the human body (43). Similarities in the expression changes of genes involved in the pathogenesis of T2DM between humans and cats deserve special attention and suggest similarities in the pathogenesis of the disease.

#### 2.1.4 Nutrition

In their natural environment, cats feed on small mammals and birds: food that is rich in protein, moderately rich in fat, and with minimal carbohydrates (19). Proteins in cats’ diet are needed to maintain blood glucose levels, as cats do not have many adaptations to regulate carbohydrate digestion. Cats do not secrete salivary amylase and have low intestinal and pancreatic amylase and intestinal disaccharidase activity (19). In addition, hepatic glucokinase activity is low and is not adaptive to increased carbohydrate consumption and the feline liver does not secrete fructokinase. Consequently, the cat’s body’s ability to minimize hyperglycemia is severely compromised when there is a large amount of glucose in the diet (19, 25). Additionally, large amounts of carbohydrates in a cat’s diet reduce the digestibility of proteins (19).

A high-protein/low-carbohydrate (HP) diet is recommended for feeding obese cats because its use affects the maintenance of normal insulin sensitivity of fat metabolism (44). The HP diet has the effect of increasing heat production and consequently facilitating fat loss (44). Cats fed an HP diet are less likely to develop obesity and show less insulin resistance compared to cats fed the same amount of calories but on a high-carbohydrate/low-protein (HC) diet (44). HC diets during obesity therapy have been shown to reduce lean body mass loss and promote fat loss (45–47). Additionally, compared to HP diets, HC diets decrease insulin sensitivity and cause hyperinsulinemia (24). As in obese cats, in cats with T2DM, the use of the HP diet is beneficial and leads to an improvement in insulin requirements (44).

The cat has a similar narrative in the “Carnivore Connection” theory as humans. This theory states that the cause of the current epidemic of obesity and diabetes in humans is potentially because, over the last two million years of evolution humans have consumed a predominantly low-carbohydrate, high-protein diet. As a result, they developed insulin resistance, which was beneficial with such a diet because it allowed them to control the balance of glucose levels and had a positive effect on fetal development. With the development of agriculture and the industrialization of the population, the diet changed, and man began to consume more cereals and starches. Populations that have recently adopted agriculture and have been on a high-carbohydrate diet for a shorter time, such as the Pima Indians, are more likely to develop T2DM, while Europeans, who have used agriculture for 12,000 years, have a lower incidence of diabetes (48). Domestic cats may have experienced a similar situation with a change from a natural, protein-rich diet to a diet consisting primarily of high-carbohydrate commercial food. Although high-carbohydrate diets lead to elevated postprandial glucose and insulin levels, and low-carbohydrate diets have been shown to improve glycemic control in cats with DM, it cannot currently be concluded with certainty that a high-carbohydrate diet is a risk factor for the development of T2DM (49).

#### 2.1.5 Obesity

The next risk factor, obesity is the condition of increased body fat mass due to too many nutrients being provided to the body than needed and may be a consequence of incorrect nutrition (20, 50). In cats, there is a strong link between obesity and diseases such as cancer, lower urinary tract diseases, especially feline idiopathic cystitis, constipation, lameness and orthopedic diseases, altered hemostasis, liver lipidosis, dermatological diseases, musculoskeletal diseases, hypertrophic cardiomyopathy, respiratory diseases, oral conditions, diabetes mellitus, atopic dermatitis, hypertension, asthma, diarrhea, ocular conditions, and allergic conditions (11, 30, 51).

Hoeing et al. (44) reported that 1 kilogram of weight gain in cats resulted in a loss of insulin sensitivity and glucose effectiveness by approximately 30% (44). Adipose tissue regulates the storage, release, degradation, and hydrolysis of fatty acids (FA). During the normal process of lipolysis, fatty acids esterify, resulting a decrease in their serum levels decrease. In obesity, there is impaired inhibition of lipolysis by insulin, resulting in increased levels of fatty acids and glycerol, and adipose tissue secretes cytokines and other pro-inflammatory molecules that antagonize the action of insulin and contribute to inflammation (52).

Additionally, the distribution of body fat in an animal is associated with insulin resistance. In cats, unlike humans, there is no gender difference in body fat distribution. Abdominal fat, which is the major site of fat accumulation in cats, is evenly distributed subcutaneously and in the abdominal cavity (44). In contrast, obese cats may deposit fatty acids into the muscle and have increased lipid levels there, which may be associated with insulin resistance (53).

It is important to note that not all obese cats develop T2DM because other genetic, environmental, and metabolic factors are involved in the pathogenesis of the disease. Obesity increases the diabetes risk by 3 to 5 times (24). However, obesity is considered to be one of the main factors in the development of T2DM and it is thought that this may be due to an increase in endogenous glucose production caused by hepatic insulin resistance, resulting in β-cell impairment and/or decreased insulin secretion due to amyloid deposition in the pancreatic islets, and as a result of β-cell failure, hypoinsulinemia and increased EGP production by the liver (54, 55).

#### 2.1.6 Insulin resistance development

In humans, obesity is thought to lead to the development of insulin resistance through a number of mechanisms. The first is the accumulation of FA in the liver and muscle as a result of abnormal FA transport and incomplete fatty acid oxidation. Additionally, oxidative stress and inflammatory response caused by adipocyte hypertrophy, including the release of pro-inflammatory cytokines resulting in elevated phosphorylation of insulin receptor substrates (iRSs). Subsequently, the secretion of adipokines by adipocytes, e.g., adiponectin increases insulin sensitivity. As the mechanism of insulin resistance in obese cats has not been studied in detail, it is presumed that its mechanisms are similar to those in obese humans. This may be indicated by reduced expression of GLUT-4 in muscle and adipose tissue, increased triglyceride content in muscle and liver, and high levels of muscle-fat ratio lipoprotein lipase expression. Unlike humans, cats (excluding the Burmese breed) do not have most of their fat stored in the abdominal cavity. In addition, insulin resistance in cats does not cause systemic inflammation as it does in humans (56).

As weight increases in the cat, insulin sensitivity and the effectiveness of insulin in lowering plasma glucose levels decrease (57, 58). As a result of the persistent state of producing excess insulin, the β-cells eventually become depleted and stop producing insulin (57). In a study by Appleton et al. (57), more than half of the cats also developed impaired glucose tolerance after gaining weight. A 30% decrease in insulin sensitivity was observed for each kilogram of excess body weight in cats (56). As mentioned, the development of insulin resistance is closely related to the development of obesity (59). This is a reversible mechanism and if the animal loses weight, the insulin resistance disappears (17, 59). Interestingly, not all obese cats develop diabetes, and the mechanisms of the obesity–insulin resistance–diabetes interaction are not yet fully understood (59). In cats with T2DM, insulin sensitivity is lower by about 6 times (17).

### 2.2 Complications of untreated type 2 diabetes in cats

The consequences of unrecognized and untreated type 2 diabetes in cats are severe. The main complications of diabetes that have been studied in cats are diabetic neuropathy and diabetic retinopathy. Diabetic neuropathy is one of the most common post-diabetic complications in cats. Although it affects 90% of individuals, only 10% of them show neurological signs. Neuropathy in cats mainly affects the pelvic limbs and motor nerve conduction, and clinical signs include hindlimb paresis, muscle atrophy most prominent in the distal parts of the pelvic limbs, plantigrade posture, difficulty or inability to jump, abnormal postural responses, ataxia, poor patellar reflex, and increased flatulence and irritability in response to touch (60, 61). Damage in nerves includes changes in Schwann cells with cleavage and ballooning of the myelin sheath, followed by demyelination, and axons of myelinated fibers involving dystrophic accumulation of axoplasmic membrane debris and glycogen, and degenerative fiber loss (61, 62). The changes observed in Schwann cells seen in cats are similar to those seen in humans with diabetes (60). Diabetic retinopathy damages blood vessels in the retina. A model of oxygen-induced retinopathy in cats was proposed as early as the 1950s, but the long development time of diabetic retinopathy and limitations in research reagents means that it is not the most popular model (63). In feline diabetics, however, diabetic cataracts often develop along with linear opacification of the posterior cortex and cortical cataracts or posterior subcapsular plaques. Complete blindness due to diabetic cataracts is very rare in adult cats, but kittens can develop more severe cataracts resulting in blindness (64, 65). Diabetic nephropathy is a serious complication of diabetes in humans, but diabetes-induced nephropathy is rare in cats (66). However, since kidney disease in older cats can be as common as diabetes, it is difficult to determine whether it is due to damage from diabetes or whether it is a comorbid condition. The incidence of chronic kidney disease in -cats ranges from 17 to 63%, and in cats newly diagnosed, 13% developed the disease during a follow-up 6-month study (67–69). In addition to the described complications, about one-third of cats with diabetes develop hypersomatotropism, resulting in the development of acromegaly. Diabetes may also be implicated in the increased development of heart disease and echocardiographic changes, but there are still few studies evaluating the impact of diabetes on heart disease in cats (70).

### 2.3 Diagnosis of type 2 diabetes in cats

Clinical signs of type 2 diabetes mellitus in cats include persistent hyperglycemia, polyuria, polyphagia, polydipsia, and decreased muscle mass with, as a consequent, weight loss, and liver enlargement (18, 22, 71).

The diagnosis of insulin sensitivity and type 2 diabetes mellitus in cats is not the same as in humans. In humans, the homeostatic model assessment (HOMA) and the quantitative insulin sensitivity method are used to assess insulin sensitivity (54). However, in cats the HOMA method is considered insufficient to reflect changes in insulin sensitivity (54). Additionally, stress-induced hyperglycemia has been observed in cats (71). A study by Ray et al. (72) showed that more than half of healthy cats without T2DM admitted to the hospital exhibited hyperglycemia (72). As cats are particularly susceptible to stress, it can be triggered by several factors, e.g., travel, being in the waiting room of a veterinary clinic, or having blood drawn for a test (71). The increase in blood glucose levels due to stress is rapid - it is observed after about 5 minutes and can last for up to 3 hours (18). Because of this unique characteristic of cats, type 2 diabetes can be diagnosed using methods that are not as influenced by stress. To diagnose a cat, it is advisable to perform a general urine test with glucose detection and relative density and a serum fructosamine determination (71). Urinary glucose occurs when serum glucose levels in cats exceed 200-288 mg/dl (11-16 mmol/l). As for the relative density of urine, it is typically 1.026-1.035 (71). Fructosamine is defined as the total of plasma proteins that have undergone the process of protein glycation. Its concentration in the blood can determine the blood glucose concentration up to 2-3 weeks before its determination. Decreased fructosamine levels are also observed when plasma protein levels are decreased and in hyperthyroidism (71). Unfortunately, the use of fructosamine is not always effective because healthy cats with naturally elevated fructosamine levels and T2DM cats with normal fructosamine levels have been reported (18). Cats with suspected T2DM may also have glycosylated hemoglobin, which indicates glucose levels approximately 70 days before the test. Additionally, this method has disadvantages, the results may be reduced in the case of anemia, and the too-long time required to reflect blood glucose concentration makes it an innefective diagnostic tool (71).

## 3 Adipokines

All substances secreted by adipose tissue are called adipokines, although the types differ. Adipokines can be hormones, such as leptin, adiponectin, plasminogen activator inhibitor-1 (PAI-1), or cytokines, such as interleukin 8 (IL-8) or tumor necrosis factor-alpha (TNFα) and chemokines (73). Among adipokines, leptin, adiponectin, resistin, and omentin, as well as cytokines and chemokines (TNF-α, IL-6), result in chronic inflammation, which may play a key role in the development of insulin resistance and T2DM and increase the risk of obesity-related cardiovascular disease, collectively referred to as the metabolic syndrome (1, 2, 74, 75).

The view of adipose tissue changed significantly after 1994, when the Ob gene and its product, leptin, were identified and characterized, and now adipose tissue is known to be an organ involved in endocrine function, the central nervous system, the immune system, and energy metabolism (8).

The observed increase in adipokines in obese patients includes cytokines, e.g., interleukin 6 (IL-6), interleukin 8 (IL-8), interleukin 18 (IL-18), TNF-α; acute phase proteins, e.g., C-reactive protein (CRP), plasminogen activator inhibitor-1 (PAI-1), haptoglobin; and chemokines, e.g., monocyte chemoattractant protein-1 (MCP-1) (16, 76). The increase in adiponectin concentration is inversely proportional to body weight gain. It has been observed that there is diurnal variation in adiponectin levels, with an increase in the morning and a decrease at night (20). Adipokines play important roles in the pathophysiology of obesity and diabetes in humans (77). There is a need to determine what role they play in T2DM in cats and whether they could be used as biomarkers of disease progression in the future (**Table 1**).

**Table 1:** Changes in levels of selected adipokines in cats and humans with T2DM and obesity compared to healthy individuals.

Adipokine	T2DM cat	T2DM human	Obese cat	Obese human
**Leptin**	↑	↑	↑	↑
**Adiponectin**	↓	↓	↓	↓
**Resistin**	ND	↑	↑	↑
**Omentin**	ND	↓	−	↓
**TNF-α**	ND	↑	↑	↑
**IL-6**	ND	↑	↑	↑

↑, increased levels; ↓, decreased levels; −, no change; ND, no data. Based on (17, 44, 58, 77–97). The similarities in the course of T2DM in humans and cats may allow us to predict how selected adipokines will change levels in T2DM in cats on the basis of data obtained in humans, but more studies are needed. Omentin, in particular, appears to be an interesting case, as it did not change the range of levels in obese cats, unlike its levels in obese humans. In the case given, one study was conducted. To obtain more data, additional studies should be conducted.

### 3.1 Leptin

Leptin is known as the central satiety signal because it regulates body composition and energy expenditure (20). It also influences sympathetic nervous system activity, hematopoiesis, and reproductive function and modulates the immune responses by having a pro-inflammatory effect and activating T cells (9, 16, 20). Additionally, is known to regulate nutritional intake, which may impact thermogenesis because it increases energy expenditure, stimulates lipolysis, and inhibits lipogenesis (24, 98). Leptin is produced by adipocytes and in minor amounts by the central nervous system (CNS), skeletal muscles, stomach, and placenta (20, 99). Although leptin has an inhibitory effect on adipose tissue cell differentiation, its secretion increases during adipocyte maturation from preadipocytes. Leptin release is initiated by TNFα and chemokines (9). High leptin levels are closely associated with high body fat mass and may lead to further weight gain and therefore the development of insulin resistance (100). Thus, along with IL-6, resistin, and TNF-α, leptin acts as an indicator of obesity initiation and insulin resistance (22).

In humans, leptin is a marker for the development of obesity, and as body fat increases, leptin levels increase (90). Additionally, it has been proposed as a biomarker for type 2 diabetes. In addition, although leptin levels increase in obese and T2DM patients, interestingly, it has been noted that diabetics with high BMI have lower leptin levels than non-diabetics with high BMI (85, 86). It has been indicated that low leptin levels have a significant effect on increasing the risk of developing insulin resistance and type 2 diabetes (86). Leptin affects the body’s insulin sensitivity by decreasing the storage capacity of triglycerides in muscle tissues and increasing fatty acid oxidation (90). Because of these unique abilities, leptin has been proposed as a potential therapeutic agent in obesity and type 2 diabetes (101).

Correlations between increased leptin levels and increased BCS and body fat mass have been demonstrated in cats (44, 58, 78–80, 87–89). Leptin levels are not dependent on the sex of the animal (88). In a study by Zapata et al. (58), diabetic cats had leptin levels 221% higher than those in normal-weight cats. Healthy cats that were obese had levels 172% higher compared to those of normal weight (58). In a study by Appleton et al. (89), lower insulin sensitivity, with higher insulin concentrations and indices, was observed in healthy, lean cats aged 1-5 years driven to overweight/obesity. Following an increased in body weight, the cats that had the highest fasting plasma leptin concentrations also had the highest fasting insulin concentrations, along with decreased insulin sensitivity (89). Increased leptin levels are also correlated with increased cat insulin resistance not only in obese but also in lean individuals (24, 89). Additionally, increased insulin levels in obese cats stimulate leptin production as in other animal species (89). As the animal’s weight decreased, serum leptin concentrations gradually decreased (81). Leptin levels in lean cats have been shown to increase with increasing age (102). It has been shown that the HC/HB diet has no effect on blood leptin levels in cats (44).

### 3.2 Adiponectin

Adiponectin is secreted by adipocytes in white adipose tissue (103). Adiponectin has anti-inflammatory effects (16). Adiponectin has several important functions, such as improving glucose and lipid metabolism, modulating insulin action, preventing inflammation, and preventing atherosclerosis (20, 104). Adiponectin receptors are located in the liver and skeletal muscle, and it has been suggested that the greatest increase in insulin sensitivity occurs in the liver. Adiponectin, together with AMP-activated protein kinase, increases glucose uptake in skeletal muscle, oxidizes fatty acids in muscle and liver, initiates the insulin signal transduction cascade in these two tissues, and increases insulin sensitivity (20, 24). Hosogai et al. (104) showed that the presence of ER stress concurrently with obesity affects the downregulation of adiponectin mRNA (104).

In humans, adiponectin levels are lower in patients with obesity, insulin resistance, and T2DM and its reduced levels can be used to predict the development of these diseases (77). Adiponectin in humans has been proposed as a biomarker for diabetes-related cardiac complications and for assessing vascular changes in peripheral type 2 diabetes. Adiponectin levels have also been proposed as a risk factor for cardiovascular disease and coronary artery disease (91). Interestingly, adiponectin has also been indicated as a target for the treatment of type 2 diabetes (77, 91, 101).

Most studies have shown that, in cats, with increasing BCS, adiponectin levels decrease (44, 78–84). However, contrary studies have also been published (82, 105, 106). Adiponectin levels in cats are not dependent on the sex of the animal (88). According to the study by Öhlund et al. (107), adiponectin levels do not differ between healthy individuals of Burmese breed, prone to developing T2DM, and Birman breed with low risk of developing DM (107). In a study by Takashima et al. (81) on healthy cats with normal BCS suffering from obesity that then lost 8% of their weight during a 9-week weight-loss period, it was shown that adipokine levels did not significantly increase in association with weight loss (81). Therefore, the hypothesis was proposed that it is not the degree of obesity that affects adiponectin levels, but other factors, such as, for example, low muscle mass (81). In the same study, a correlation between adiponectin and insulin levels was not found (81). Zapata et al. (58) showed, that compared to healthy cats of normal weight, cats with diabetes had 61% lower adiponectin levels, and cats with diabetes had 45% lower levels compared to healthy obese cats. High molecular-weight (HMW) adiponectin multimers, which are associated with insulin sensitivity, account for approximately 80% of adiponectin in cats, whereas they only account for 30% in humans (17). It has been shown that the HC/HB diet had no effect on blood adiponectin levels in cats (44).

### 3.3 Resistin

Because resistin’s mRNA has been detected in humans in circulating mononuclear cells, not adipocytes, and is released from non-fat adipose tissue cells, Fain (3) proposed that resistin is a cytokine secreted by cells of the immune system (3). Resistin is insulin-resistant and impairs glucose homeostasis. Its function is to downregulate inflammation in macrophages, peripheral blood mononuclear cell (PBMC) and vascular cells (108).

Resistin levels are elevated in obese humans, and also in humans with T2DM, but there are no data for cats with T2DM (91). Resistin is involved in the development of insulin resistance and T2DM, and for this reason, it too has been proposed as a biomarker for these diseases. In addition, in humans, it has also been proposed as a biomarker for diabetes complications related to chronic periodontitis and cardiovascular disease (91, 109).

It has been reported that resistin levels increase with increasing BCS and body fat mass in cats (78, 79). Cats with obesity have higher resistin mRNA expression in adipose tissue compared to lean cats (78). In addition to adipose tissue, in cats resistin expression may also occur in the lung and spleen (81). However, in the previously described study by Takashima et al. (81), resistin levels also did not change significantly. Resistin was negatively correlated with insulin in the blood of the cats studied (81), which is confusing, since an important role in the pathogenesis of insulin resistance and T2DM has also been indicated (79).

### 3.4 Omentin

Omentin, although not yet studied in cats with T2DM, deserves consideration because of its unique properties. It has regulatory functions, modulates insulin secretion and insulin sensitivity, and can increase insulin action, affecting fat accumulation, adverse fat breakdown, and inflammation (55, 110). In humans, omentin-1 levels are lower in individuals with T2DM compared to healthy controls (110).

In people with obesity and T2DM, omentin levels are lower than in healthy people, but not in patients with type 1 diabetes (91, 92). Omentin has anti-inflammatory, anti-obesity and anti-diabetic properties, and these are indicated when omentin is proposed as a therapeutic target (90, 91). Omentin has also been proposed as a marker for predicting obesity and T2DM risk (90).

In cats, Williams et al. showed that blood concentrations of omentin are not dependent on BCS, but are 56% higher in males (88). In cats, omentin levels were also not correlated with insulin or glucose levels, as has been reported in humans (88).

### 3.5 TNF-α

TNF-α has various important functions related to adipose tissue regulation, it responds to inflammation, induces apoptosis, stimulates lipolysis, modulates insulin sensitivity, induces insulin resistance, inhibits adipocyte differentiation, disrupts leptin homeostasis, and induces chemokine secretion (9, 16). TNF-α stimulates IL-6 and MCP-1 expression and inhibits adiponectin mRNA expression. Wang et al. (16) described the lack of effect of TNF-α on leptin expression in humans (16). Among the roles of TNF-α are also the promotion of lipid mobilization, inhibition of adipocyte differentiation, and reduction of lipid storage capacity (79).

In humans, TNF-α levels are elevated in people with T2DM and obesity, and TNF-α itself plays roles in the development of insulin resistance (93). TNF-α and its receptors have been indicated to be biomarkers for the progression of diabetic kidney disease (111, 112) and for assessing the degree of diabetic retinopathy (93).

In cats, TNF-α levels increase with increasing BCS and body weight, and increased levels of TNF-α expression have been shown in adipose tissue and skeletal muscle in obese cats (94, 95). However, in a study by Hoenig et al. in cats that were driven to obesity, TNF-α levels did not show significant changes (80).

### 3.6 IL-6

IL-6 is a pro-inflammatory cytokine secreted from leukocytes, endothelial cells, and adipocytes. Its plasma levels in obese patients are directly proportional to weight loss and fat loss. It regulates the development of chronic diseases such as cardiovascular disease (CVD), where it induces the secretion of a risk marker, hepatic C-reactive protein, and type 2 diabetes mellitus (113). By decreasing lipoprotein lipase (LPL) activity, it increases blood lipid levels (113). By inhibiting the expression of the insulin receptor, insulin receptor substrate-1, and glucose transporter type 4 in 3T3-L1 adipocytes, IL-6 suppresses insulin activity (113) A correlation has been shown between increased IL-6 levels and the occurrence of insulin resistance (3). Additionally, attenuating adiponectin mRNA synthesis and secretion, and inhibition of insulin signaling in hepatocytes contribute to insulin resistance (113).

In humans, IL-6 levels increase in patients with T2DM and obesity (96, 97). IL-6 has been shown to be correleted with the development of complications in diabetes, including diabetic neuropathy, in which high levels of IL-6 are associated with increased nerve degeneration (90, 114).

Plasma IL-6 levels are elevated in obese cats (17). Here again, Hoeing et al. showed no significant changes in IL-6 levels in cats that had gained weight (80).

## 4 Conclusion

T2DM is a serious disease that an increasing number of domestic cats are suffering from. The multitude of factors that can cause this disease indicate its severity. Some of the risk factors, such as nutrition and the development of obesity, can be influenced by the pet’s caregiver. Therefore, it is worthwhile for veterinary professionals to educate cat guardians on the proper management of the animal to limit the development of the disease.

The domestic cat is still a very poorly studied species in terms of the pathogenesis of diabetes mellitus. Most of the information on the subject is still based on data obtained from humans. This is enabled by the high similarity of feline diabetes to human T2DM. In order to better understand the exact mechanisms of the disease, especially regarding adipokines, studies focusing not only on obesity, which is indeed also an important factor in the development of T2DM, but also on cats already suffering from diabetes themselves, are needed.

The data collected in the above review indicate a promising role for the presented adipokines as biomarkers of type 2 diabetes in cats. Adipokines may be a promising means to control disease progression and recovery in the animal. Notably, adipokines have been proposed as biomarkers of disease in humans, e.g., resistin as a biomarker for diabetes complications related to chronic periodontitis and cardiovascular disease. Biomarkers are useful in the course of disease progress, as they can be used not only for risk assessment and screening, but also to determine the severity of the disease and to select and monitor therapy (109). It is particularly important to discover rapid, sensitive and effective methods for risk assessment in feline type 2 diabetes.

## Author contributions

Conceptualization, OS and PN-R. Methodology, OS and PN-R. Software, OS. Validation, OS. Formal analysis, OS. Investigation, OS and PN-R. Resources, PN-R. Data curation, OS. Writing—original draft preparation, OS. Writing—review and editing, OS and PN-R. Visualization, OS. Supervision, PN-R. Project administration, PN-R. Funding acquisition, PN-R. All authors have read and agreed to the published version of the manuscript.

## Conflict of interest

The authors declare that the research was conducted in the absence of any commercial or financial relationships that could be construed as a potential conflict of interest.

## Publisher’s note

All claims expressed in this article are solely those of the authors and do not necessarily represent those of their affiliated organizations, or those of the publisher, the editors and the reviewers. Any product that may be evaluated in this article, or claim that may be made by its manufacturer, is not guaranteed or endorsed by the publisher.
